# Correlation between the −1562C/T polymorphism in the matrix metalloproteinase-9 gene and hemorrhagic transformation of ischemic stroke

**DOI:** 10.3892/etm.2015.2186

**Published:** 2015-01-16

**Authors:** XIAOMAN ZHANG, XINHUI CAO, XIAOYU XU, AIFAN LI, YUMING XU

**Affiliations:** 1 Department of Neurology, The First People’s Hospital of Zhengzhou, Zhengzhou, Henan 450004, P.R. China; 2 Department of Neurology, The First Affiliated Hospital of Zhengzhou University, Zhengzhou, Henan 450052, P.R. China

**Keywords:** matrix metalloproteinase-9, gene polymorphism, hemorrhagic transformation

## Abstract

The aim of the present study was to investigate the correlation between the −1562C/T polymorphism in an intron of the matrix metalloproteinase-9 (MMP-9) gene and hemorrhagic transformation of ischemic stroke (IS). Using polymerase chain reaction-restriction fragment length polymorphism, the −1562C/T polymorphisms in 222 patients with IS were detected. The patients were divided into hemorrhagic transformation (HT; 84 cases) and non-hemorrhagic transformation (NHT) groups (138 cases) depending on the results from the susceptibility-weighted magnetic resonance imaging, which was performed between one and two weeks following stroke onset. The allele frequencies were subsequently compared. Baseline data of the two groups were comparable. The HT group exhibited a significantly lower frequency of the CT+TT genotype compared with the NHT group (17.86 vs. 30.43%, P<0.05). In addition, the frequency of T allele was significantly lower in the HT group compared with the NHT group (8.93 vs. 15.94%, P<0.05). Therefore, the results indicated that the −1562C/T polymorphism in the MMP-9 gene is correlated with hemorrhagic transformation of IS in the population studied. Furthermore, the T allele may be a protective factor for hemorrhagic transformation of IS in this population.

## Introduction

Cerebrovascular disease is the leading cause of mortality in China, and ischemic stroke (IS) accounts for 70% of these cases. Previous studies have demonstrated that thrombolytic therapy is effective at alleviating symptoms of these patients if performed between 3 and 6 h following the stroke; however, this treatment typically increases the risk of patient hemorrhage (1,2). Thus, techniques are required to identify patients with a high risk of hemorrhagic transformation following thrombolytic therapy for IS.

Matrix metalloproteinase (MMP)-9, a gelatinase and a major regulator of the extracellular matrix, can degrade major components of the cerebral vascular basement membrane, and is considered to be an indicator of an increased risk of atherosclerotic plaque development (3). In a previous preliminary study, the association between MMP-9 and hemorrhagic transformation of IS was systemically investigated, and a significant correlation was observed. Furthermore, a previous study revealed that MMP-9 may be an indicator of unstable plaque formation and rupture in patients with cerebrovascular disease (4). Polymorphisms of the MMP-9 gene have been shown to significantly impact the concentration and activity of MMP-9. For example, certain changes in the promoter sequence of MMP-9 can lead to increased expression of MMP-9. Pöllänen *et al* confirmed the correlation between the −1562C/T polymorphism of the MMP-9 gene and the instability and rupture of atherosclerotic plaques (5).

In the present study, the correlation between the MMP-9 gene −1562C/T polymorphism and hemorrhagic transformation was analyzed, with the aim of further characterizing the mechanism of hemorrhagic transformation following IS.

## Subjects and methods

### Subject selection

Subjects were recruited from the six wards of the Department of Neurology, The First People’s Hospital of Zhengzhou (Zhengzhou, China) between December 2011 and February 2013. The study population included 84 cases of hemorrhagic transformation of IS (HT group) and 138 cases of acute IS without hemorrhagic transformation (NHT group). The two groups were of comparable age and gender composition, and all subjects were first time ischemic cerebrovascular disease patients, native to Henan province. Patients were excluded if they had any history of severe liver, kidney or endocrine disease, a coagulation disorder or in the case of pregnancy in female patients. All cases in the NHT group, including 67 male and 71 female patients aged 62.58±7.84 years, were diagnosed according to the ‘Diagnostic criteria for cerebrovascular disease (1995)’ revised on the Fourth National Conference on Cerebrovascular Disease by the Chinese Academy of Neurology (6), and confirmed by magnetic resonance imaging. All acute IS patients underwent susceptibility-weighted imaging (SWI) 7–14 days after stroke onset. There was no statistically significant difference in the mean span between the two groups (8.46±1.01 vs. 8.2±1.86 days in the HT and NHT groups, respectively; P>0.05). The HT group consisted of 84 patients, including 44 male and 40 female patients with a mean age of 60.86±8.72 years. There was no statistically significant difference in mean age between the two groups (P>0.05). The National Institutes of Health Stroke Scale was used for quantitative assessment of IS severity in all patients and a comparison of the mean scores between the two groups revealed no statistically significant difference (11.25±3.12 vs. 10.68±2.95 in the HT and NHT groups, respectively; P>0.05). Patients received anti-platelet therapy according to their general pathogenic condition; however, patients who required anticoagulative or thrombolytic therapy were excluded from the study. SWI was used to calculate the areas of infarction. There were no statistically significant differences observed between the two groups with regard to the patient baseline characteristics (Table I). This study was conducted in accordance with the Declaration of Helsinki and with the approval from the Ethics Committee of the First People’s Hospital of Zhengzhou. Written informed consent was obtained from all the participants.

### Extraction of genomic DNA

A 5-ml peripheral blood sample was collected from each patient with an EDTA tube, and genomic DNA was extracted using the classic phenol-chloroform-proteinase K method (7). The DNA was then dissolved with 200 μl Tris-EDTA buffer and stored at −20°C in aliquots for future use.

### Polymerase chain reaction (PCR) amplification

Primers were synthesized by Beijing Dingguo Biotechnology Co., Ltd. (Beijing, China), which targeted the −1562C/T polymorphism of MMP-9; the sequences were as follows: Forward, 5′-GCCTGGCACATAGTAGGCCC-3′ and reverse, 5′-CTTCCTAGCCAGCCGGCATC-3′. A 15-μl reaction system was produced by sequentially adding 7.5 μl master mix (Thermo Fisher Scientific, Waltham, MA, USA), 6.0 μl ddH_2_O, 1 μl DNA template, and 0.25 μl each of the forward and reverse primers to a PCR tube. The PCR conditions were as follows: Predenaturation at 94°C for 2 min; 25 cycles of 94°C for 30 sec, 55°C for 30 sec and 72°C for 45 sec; followed by extension at 72°C for 10 min. A 3-μl PCR product was verified by agarose gel electrophoresis (Eagle Eye II; Agilent Technologies, Inc., Santa Clara, CA, USA).

### Restriction enzyme digestion of the PCR product

A 10-μl reaction system was produced by sequentially adding 5.8 μl ddH_2_O, 3 μl PCR product, 1 μl loading buffer and 0.2 μl *Sph*I restriction enzyme (Fermantas, Pittsburgh, PA, USA). The 10 μl reaction system was incubated in a thermocycler (9700; Applied Biosystems Life Technologies, Foster City, CA, USA) at 37°C for 6 h. Following termination of the reaction, a 10-μl sample of the product was analyzed by agarose gel electrophoresis.

### Statistical analysis

Statistical analysis was performed with SPSS 16.0 software (SPSS, Inc., Chicago, IL, USA). The numbers of each genotype were counted and the allele frequency was calculated for the two groups. Representativeness of sample was verified using the Hardy-Weinberg equilibrium test, and genotype or allele frequencies were compared using the χ^2^ test. False positive rate α was set to 0.05, and a bilateral probability of P≤0.05 was considered to indicate a statistically significant difference.

## Results

### PCR-restriction fragment length polymorphism for the MMP-9 polymorphism

**Figure 1:**
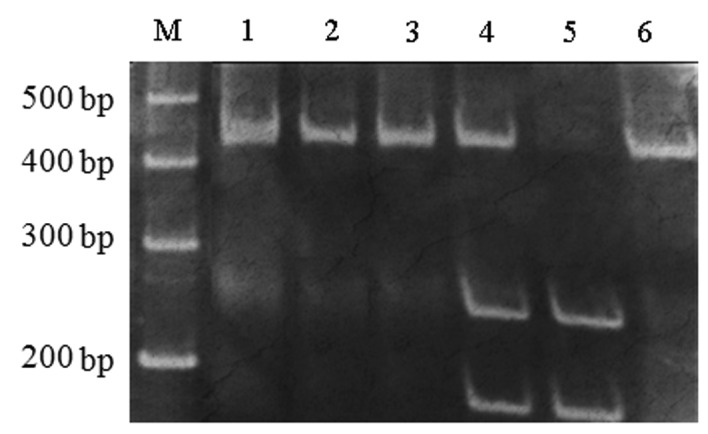
Restriction enzyme digestion of the matrix metalloproteinase-9 polymerase chain reaction (PCR) products. Lanes: 1, 2 and 3, PCR products; 4, CT genotype; 5, TT genotype; 6, CC genotype; M, DNA marker.

The PCR product of each sample was separated by agarose gel electrophoresis, and a 435-bp band was observed. Digested PCR products showed one of the following patterns: A single 435-bp band (CC homozygotes), 247- and 188-bp bands (TT homozygotes), and 435-, 247- and 188-bp bands (CT heterozygotes; Fig. 1).

### Hardy-Weinberg equilibrium test

Numbers of each genotype at the MMP-9 gene −1562C/T polymorphism site showed no statistically significant difference compared with the expected value (P>0.05), indicating that the selected samples were obtained from a population in genetic equilibrium and were representative (Table II).

### Comparison of genotype and allele frequencies

Three genotypes were identified in the study. The TT genotype was rare, with no cases observed in the HT group and only two observed in the NHT group. The frequency of the CT+TT genotype was significantly lower in the HT group compared with that in the NHT group (P<0.05; Table III), as was the frequency of the T allele (P<0.05; Table IV).

### MMP-9 −1562C/T polymorphism for risk assessment of hemorrhagic transformation of IS

The relative risk of hemorrhagic transformation was compared among the three genotypes and between the two alleles. The results revealed that the odds ratio (OR) of the CT+TT genotype was 0.497 [95% confidence interval (CI), 0.255–0.967], while the OR of the T allele was 0.517 (95% CI, 0.278–0.961). Therefore, T allele carriers and homozygotes exhibited a significantly lower risk of hemorrhagic transformation following IS.

## Discussion

Cerebrovascular disease is the leading cause of mortality in China, and IS accounts for 70% of these cases. The most effective treatment for IS is thrombolytic therapy that is performed within 3–6 h after stroke onset. Although patients may benefit from this therapy, a number of individuals suffer an increased risk of hemorrhagic transformation. A meta-analysis of previous studies indicated that the MMP-9 gene may be an independent risk factor for hemorrhagic transformation (8). Previous studies have demonstrated that MMP-9 is closely associated with vascular damage, as its specific collagenase and elastase activity can degrade a number of components of the extracellular matrix (9,10). In addition, MMP-9 is responsible for the destruction and reconstruction of related tissues underlying the vascular endothelium. To date, studies on MMP-9 polymorphisms have indicated an association with a number of tumors, cardiovascular disease, autoimmune diseases, schizophrenia and stomatitis (11,12). Furthermore, the upregulation of MMP-9 was revealed to be involved in the development and progression of pituitary tumor hemorrhage (13). A previous study also demonstrated a correlation between MMP-9 and hemorrhagic transformation following infarction (14), while increased expression of MMP-9 has been associated with intraplaque hemorrhage in a swine model of vulnerable carotid atherosclerosis (15).

In the human genome, the MMP-9 gene is located on the long arm of chromosome 20 (20q11.1–13.1), and its expression is regulated mainly at the transcriptional level. The −1562C/T polymorphism in the MMP-9 promoter may reduce the rate of transcription by inhibiting protein binding, which downregulates MMP-9 expression. Thus, the −1562C/T polymorphism is associated with the development of a number of diseases. A previous study on a Polish population revealed that the −1562C/T polymorphism in the MMP-9 gene is significantly correlated with ischemic heart disease (16). However, the study by Szczudlik and Borratyńska identified no correlations between the −1562C/T polymorphism and infarction, subarachnoid hemorrhage or spontaneous intracerebral hemorrhage (17). Pöllänen *et al* revealed that the −1562C/T polymorphism of the MMP-9 gene positively correlated with atherosclerotic plaque instability and plaque rupture (5). Furthermore, an additional study identified that increased expression levels of MMP-9 were associated with intraplaque hemorrhage in a swine model of vulnerable carotid atherosclerosis (15). However, other studies have hypothesized that MMP-9 deficiency is protective against hemorrhagic transformation following the early stages of ischemia and reperfusion, and genetic variations in the MMP-9 gene are not associated with hemorrhagic transformation occurrence in patients treated with tissue-type plasminogen activator (18). In China, previous studies have indicated that the −1562C/T polymorphism is not clearly correlated with coronary atherosclerosis in the Chinese Han population, but is significantly correlated with acute coronary syndrome (19). Furthermore, the T allele may be an important risk factor of aortic dissection in hypertensive patients of this ethic origin (20). Moreover, a correlation between the −1562C/T polymorphism and abdominal aortic aneurysm has been demonstrated by Duellman *et al* (21). In summary, the role of the MMP-9 gene −1562C/T polymorphism in the development of numerous diseases remains controversial, which may be due to geographical and ethnic differences.

To the best of our knowledge, the present study is the first to investigate the correlation between the −1562C/T polymorphism of the MMP-9 gene and hemorrhagic transformation of acute IS in a Han population from Henan Province. In the study, the selected sample population was in Hardy-Weinberg equilibrium; thus, was representative. The TT genotype was scarce at the −1562C/T polymorphism site of the MMP-9 gene, which was consistent with the results obtained in Han populations from other areas of China (22,23). Furthermore, genotype and allele frequencies at the −1562C/T polymorphism site showed statistically significant differences between the two groups. Therefore, it was hypothesized that the −1562C/T polymorphism may be associated with hemorrhagic transformation following IS in the Han population from Henan Province, and that the T allele may be a protective factor for this condition.

## Figures and Tables

**Table I:** Baseline characteristics of the two groups.

Items	HT group	NHT group	t-value	P-value
Age (years)	60.86±8.72	62.58±7.84	1.519	0.126
Gender, male/female (n)	44/40	67/71	0.678	0.339
SBP (mmHg)	158.21±28.14	152.36±27.83	1.513	0.126
DBP (mmHg)	90.19±18.37	89.46±17.51	0.296	0.758
NIHSS score	11.25±3.12	10.68±2.95	1.366	0.194
SWI span (days)	8.46±1.01	8.2±1.86	1.179	0.175
Ischemic areas (cm^2^)	3.01±1.12	2.98±0.97	0.211	0.786
Infarction, cardiogenic/non-cardiogenic (n)	8/76	14/124	0.023	0.881

HT, hemorrhagic transformation; NHT, non-hemorrhagic transformation; SBP, systolic blood pressure; DBP, diastolic blood pressure; NIHSS, National Institute of Health Stroke Scale; SWI, susceptibility-weighted imaging.

**Table II:** Hardy-Weinberg equilibrium test for the MMP-9 gene polymorphism in the HT and NHT groups.

	HT group		NHT group	
		
Genotype	Observed value	Expected value	Observed value	Expected value
CC	69	69.7	96	97.5
CT	15	13.7	40	37.0
TT	0	0.7	2	3.5

HT group: χ^2^=0.807, P=0.369; NHT group: χ^2^ =0.917, P=0.338. HT, hemorrhagic transformation; NHT, non-hemorrhagic transformation; MMP-9, matrix metalloproteinase-9.

**Table III:** Comparison of genotype frequency between the two groups.

Group	CC genotype, n (%)	CT+TT genotype, n (%)	χ^2^	P-value
HT	69 (82.14)	15 (17.86)	4.329	0.037
NHT	96 (69.57)	42 (30.43)		

HT, hemorrhagic transformation; NHT, non-hemorrhagic transformation.

**Table IV:** Comparison of allele frequency between the two groups.

Group	C allele, n (%)	T allele, n (%)	χ^2^	P-value
HT	153 (91.07)	15 (8.93)	4.458	0.035
NHT	232 (84.06)	44 (15.94)		

HT, hemorrhagic transformation; NHT, non-hemorrhagic transformation.
